# Changes in Age Stereotypes in Adolescent and Older Participants of an Intergenerational Encounter Program

**DOI:** 10.3389/fpsyg.2021.658797

**Published:** 2021-04-16

**Authors:** Dirk Kranz, Nicole Maria Thomas, Jan Hofer

**Affiliations:** Department of Psychology, Trier University, Trier, Germany

**Keywords:** intergenerational programs, evaluation, adolescents, older adults, age stereotypes, ageism, youthism

## Abstract

This intervention study explored the effects of a newly developed intergenerational encounter program on cross-generational age stereotyping (CGAS). Based on a biographical-narrative approach, participants (secondary school students and nursing home residents) were invited to share ideas about existential questions of life (e.g., about one’s core experiences, future plans, and personal values). Therefore, the dyadic Life Story Interview (LSI) had been translated into a group format (the Life Story Encounter Program, LSEP), consisting of 10 90-min sessions. Analyses verified that LSEP participants of both generations showed more favorable CGAS immediately after, but also 3 months after the program end. Such change in CGAS was absent in a control group (no LSEP participation). The LSEP-driven short- and long-term effects on CGAS could be partially explained by two program benefits, the feeling of comfort with and the experience of learning from the other generation.

## Introduction

Developing and maintaining a positive image of people of different age groups is vitally important to society as well as to the individual. Young individuals with a positive image of older people are more likely to benefit from older people as mentors and models, and they are less anxious about their own aging (Allan and Johnson, 2008; Zucchero, 2011). Older individuals with a positive image of younger people have more intergenerational contact; they receive more support from the young and engage more in generative behavior, which is closely associated with the experience of meaning and happiness (Cheng, 2009; Hofer et al., 2014).

Against this backdrop and considering constant increase in life expectancy and fundamental changes in family structure (e.g., smaller family sizes, larger geographic distances between parents and their adult children, and professionalization of support arrangements), it is not surprising that there is growing interest in and public promotion of intergenerational programs (for an overview, see Kaplan and Sánchez, 2014). According to the UNESCO Institute for Lifelong Learning, intergenerational programs are “social vehicles that create purposeful and ongoing exchange of resources and learning among older and younger generations” (Kaplan, 2001, p. 4).

In the present study, we explored whether and to what extent participating in a newly developed intergenerational program with a focus on individual life stories and existential life issues had an impact on cross-generational age stereotyping (CGAS). Our intervention study is innovative in that it systematically evaluated a theoretically grounded program, tailored to adolescents and older people, with regard to young participants’ image of older people as well as older participants’ image of young people.

### Age Stereotypes and Intergenerational Contact

In general, stereotypes are overgeneralized beliefs about people who belong to a particular social group, such as people of a particular sex, race, or age. Stereotypes often underlay prejudice and discrimination, as in the case of sexism, racism, and ageism. Although the latter term was initially coined to describe young individuals’ negative beliefs about older people (Butler, 1969), it can be equally used to describe negative beliefs that older individuals have about younger people (cf. Kite and Johnson, 1988; Kite et al., 1991). Stereotypes are not necessarily negative; many include positive aspects as well. Age stereotypes are especially ambivalent. For example, young individuals stereotypically perceive the older generation as warm but incompetent (Cuddy et al., 2005), while older individuals perceive the young generation as open-minded but foolish (Hummert et al., 2004). Overall, however, the old age stereotype seems to be more negative than the young age stereotype, mirroring the idealization of youthfulness in contemporary Western societies (see Hummert, 2011, for an overview).

Like a vicious circle, an unfavorable view of “the old” or “the young,” respectively, inhibits intergenerational contact, which, in turn, reinforces negative CGAS (Hagestad and Uhlenberg, 2005; Abrams et al., 2006). Therefore, one of the most efficient ways to reduce negative age (or any other) stereotypes is to promote contact across intergroup boundaries. In his pioneering work on “the nature of prejudice,” Allport (1954) suggested that it is not sufficient to create contact alone, but that a set of interaction conditions have to be met to overcome negative attitudes toward an outgroup. These conditions include common goals, intergroup cooperation, equal group status, and institutional support. A meta-analysis of more than 500 studies by Pettigrew and Tropp (2006) powerfully supported the contact hypothesis, corroborating a robust and positive impact of contact on intergroup attitudes, thereby suggesting that the conditions of Allport (1954) should be considered facilitating rather than necessary.

Based upon the contact hypothesis, numerous intergenerational programs were established in recent years that aimed to reduce ageism. Reviews of these programs largely confirmed positive effects and identified several moderating factors: the meaningfulness of activities to both age groups, mutual attention and confidence (Giraudeau and Bailly, 2019); the experience of personal well-being and learning from one another (Martins et al., 2019); and the inclusion of educational elements and demographics, with stronger program effects for female compared to male participants and adolescents compared to children (Burnes et al., 2019). These reviews also pointed to limitations of many intergenerational programs: a lacking theoretical rationale for the program contents (Kuehne and Melville, 2014) and no rigorous evaluation research, including a biased focus on program effects on young participants (Martins et al., 2019; Lee et al., 2020). To our knowledge, only four studies examined stereotype change for both young and older participants of intergenerational programs: Gamliel and Gabay (2014) and Meshel and McGlynn (2004) found positive age stereotyping effects for both age groups, whereas Belgrave (2011) and Pinquart et al. (2000) found positive effects for older participants’ young age stereotypes, but not vice versa. Strikingly, the former two programs included educational elements, whereas the latter two programs consisted of leisure activities only.

### The Life Story Encounter Program

Our interest concerned changes in CGAS through a new intergenerational encounter program. We developed a program, namely, the Life Story Encounter Program (LSEP), that provides adolescents and older people the opportunity to address existential questions of life (e.g., “Who am I?,” “Where do I come from?,” and “What do I live for?”) on the basis of biographical memories. We decided for such a narrative approach since individuals make sense of the world and the self through the stories they live by – and share (Ricoeur, 1991; Gergen, 1994). Biographical-narrative elements are included in almost all counseling and psychotherapy, especially in the gerontological field. Specific methods include, for example, biographical work and reminiscence therapy (Lin et al., 2003; Bornat, 2008). There exist a number of evaluated biographical-narrative intergenerational programs (for an overview, see Park, 2015). These programs are characterized by unidirectional communication between the generations (i.e., older participants’ storytelling to young children); and the evaluation research is mostly qualitative, with a focus on older participants’ psychosocial wellbeing or young participants’ prosocial behavior (e.g., in family or school).

Regarding the LSEP, we translated the dyadic Life Story Interview (LSI) by McAdams (2007) into a group format. The LSI is a well-established semi-structured procedure, wherein an interviewer asks the participant a series of questions designed to elicit 10 core features of their self-defining life story, such as core experiences, future plans, and personal values. Participants of the LSEP are asked to commonly reflect upon and talk about these features, as further described in Table 1. Importantly, the communication structure of the LSEP is not unidirectional, as is the case in the LSI and most existing narrative intergenerational programs, but bidirectional. Reciprocity has been shown to be crucial for the acceptance and effectiveness of intergenerational programs in adolescent participants (Mannion, 2012; Knight et al., 2014).

**Table 1:** Overview of the 10 Life Story Encounter Program (LSEP) sessions.

Session number and focus^*^	Core questions	Icebreaker
1. Life chapters (A)	What are the main chapters in your personal book of life? How did you get from one chapter to the next?	Picture: Life Stages (a prominent theme in 19th century art, conveying the idea of a human’s life being divisible into stages – up and down, and mostly with young adulthood at the top).
2. High, low, and turning points (B1–B3)	What positive or negative episode stands out in your life? What episode has marked an important change in your life?	Picture: Mountainous landscape (high mountains and deep valleys as allegories of the ups and downs of the life course).
3. Life memories (B4–B6)	What is a very positive, happy memory or a very negative, unhappy memory from your childhood, youth, and adulthood?	Symbol: Madeleines (small French sponge cakes that made Marcel Proust, 1871–1922, in his “A la recherche du temps perdu,” nostalgic childhood memories arise). Participants are offered to taste madeleines.
4. Life experience and wisdom (B7)	What is an episode in your life in which you displayed wisdom? What does this memory say about you and your life experience?	Pictures: Mother Teresa (1910–1997), Emma Watson (*1990), Nelson Mandela (1918–2013), and Socrates (470–399).
5. Religion and spirituality (B8)	Is there an episode or moment in which you felt something Devine, or some ultimate force, or a feeling of oneness with nature, the world, or the universe?	Story: “Mittagessen mit Gott” (“Lunch with God”; a short story about a little boy and an older woman, having a chance encounter and an amazing lunch break in a park).
6. Future plans (C1–C3)	What do you see to be the next chapter in your life? What are your plans, dreams, or hopes for the future?	Song: “Für mich soll’s rote Rosen regnen” (“It should rain red roses for me”; an optimistic song about aging by Hildegard Knef, 1925–2002, a well-known German cabaret singer).
7. Life challenges (D1–D3)	Looking back over your life, how have you coped with health problems, interpersonal conflicts, and losses of loved ones?	Picture: “Eddie the Eagle” (Michael Edwards, *1963, the first but hopeless competitor since 1928 to represent Great Britain in Olympic ski jumping in 1988).
8. Failure and regret (D4)	How have you coped with failures or regrets? What effects have these failures or regrets had on you and your life?	Song: “Non, je ne regrette rien” (“No, I regret nothing”; a bittersweet song about failure, regret, and self-acceptance by Edith Piaf, 1915–1963, the most widely known French chanteuse).
9. Personal values (E1–E4)	What are your religious beliefs and values, your political and social views? What is the most important value in human living?	Symbol: Compass (as the compass indicates orientation, so personal values are landmarks that are fundamental to navigating one’s life). Each participant gets a little compass.
10. Life themes (F, G)	Looking back over your life story, do you discern a central theme, message, or idea that runs throughout the story?	A flipchart paper from the first session appears once again; it summarizes participants’ variety in dividing the life course into major stages or periods.

* Letters/numbers in parenthesis indicate the corresponding chapters in the Life Story Interview (LSI; McAdams, 2007).

LSEP groups consist of about five adolescents, five older persons, and, ideally, two moderators. The participants meet once a week over a period of 10 weeks, with each session lasting about 90 min. An LSEP meeting typically starts with the moderators’ welcome and introduction into the session topic. An icebreaker then leads into the group talk. Icebreakers are, for example, “Life Stages,” a prominent theme in 19th century art, in the session about life chapters, or Edith Piaf’s chanson “Non, je ne regrette rien” in the session about failure and regret (see Table 1 again). The moderators interfere only if any of the group members digress from the session topic, show all too strong emotions, dominate or completely withdraw from the talk, or in case of heavily unbalanced shares in speech between the generations. At the end, the participants are asked about a session review, and the moderators give an outlook to the next session. The first and last sessions differ from the others in that they include an introduction of participants and program and a little farewell party and group photo shooting, respectively. The LSEP handbook (Hofer et al., 2020), describing the program in comprehensive detail, can be obtained in a digital form from the first author.

The LSEP seeks to consider the four conditions of positive intergroup contact by Allport (1954). Based on their specific individual histories, the adolescent and older participants are invited to share their thoughts about existential questions of life. The goal is not to reach a consensus but rather to find out which answers are common across generational lines and which are idiosyncratic. Although the older participants might be especially concerned with guiding the next generation and adolescents with forming their identity (Erikson, 1959), there is no program inherent expert-novice dichotomy between the generations. Finally, program participation should be ideologically and logistically supported by participants’ institutions (e.g., secondary schools and nursing homes).

The contact hypothesis is about *when* intergroup contact reduces negative outgroup attitudes. We were additionally interested in specific processes at work, that is, in *how* CGAS changes within intergenerational programming. Referring to the seminal group interaction theory by Bales (1950), two fundamental group processes might be relevant: social-emotional and task-related processes. With regard to the LSEP, the former includes feelings of comfort with members of the other generation during the 10 group sessions, while the latter includes learning from one another. Bales (1950) proposed, and verified by small group observation, that both social-emotional and task-related processes are essential and need to be well-balanced for a positive group development and performance. A group without a feeling of unity and cohesion risks breaking apart, while a group without a common task or goal to accomplish risks losing its dynamic (see Keyton, 2018, for a review).

### Objectives and Hypotheses

Using a longitudinal design including a nonrandomized control condition, our study aimed to examine the impact of participating in the newly developed LSEP on CGAS. Importantly, the LSEP goes beyond a pure leisure program; using a biographical-narrative approach, it tries to initiate a genuinely meaningful dialog between adolescents and older people. We propose the following three hypotheses about the effects of LSEP participation on age stereotyping:

1. Given the general idealization of youthfulness in contemporary Western societies, the baseline age stereotype of young people is more positive than that of older people, irrespective of participants’ age.
2. Referring to the contact hypothesis by Allport (1954), the LSEP promotes a more positive stereotype of the other generation, namely, a positive change in young participants’ old age stereotype and a positive change in older participant’s young age stereotype alike.
3. Referring to the group interaction theory by Bales (1950), this positive cross-generational stereotype change can be explained by two benefits of the program, namely, participants’ social comfort and their learning experience during the LSEP.

## Materials and Methods

### Participants

Study participants were secondary school students (young participant group) and nursing home residents (older participant group). Both their institutional contexts are highly age-segregated, with little extraprofessional and/or extrafamilial contact between the generations. The initial sample consisted of *N* = 606 participants: *n* = 404 students (50% female; 14–20 years, *M* = 16.29, *SD* = 0.90) and *n* = 202 residents (71% female; 64–98 years, *M* = 84.37, *SD* = 7.07). All participants were from a medium-sized city and surroundings in the western part of Germany. The students came from secondary schools (all Gymnasium type; school track with the highest level of education in Germany); the older participants lived in various nursing homes. About half of the older participants had a lower educational level (Volksschule: 55.0%), others were medium (Realschule: 21.3%) or highly educated (Gymnasium: 23.8%).

Older participants had been preselected by nursing home staff members based on two criteria: age (over 60 years) and health (no bedfast and no severe cognitive, auditory, or speech impairments). We additionally applied a dementia screening at the first measurement point, the Dementia Detection Test (DemTect; Kalbe et al., 2004), consisting of five tasks: an immediate and delayed recall of a word list, a number transcoding task, a word fluency task, and a digit span reverse. Deviating from the nursing home staff’s assessment, this screening revealed that only 42% of the older participants in the program condition showed no cognitive impairment; 40% showed mild cognitive impairment; and 18% were suspected of early dementia. Nevertheless, we trusted in the staff’s practical knowledge based on daily interactions with the residents and did not exclude anyone based on the DemTect screening result.

A subsample of the initial sample followed our invitation to participate in the LSEP (“program group”): *n* = 59 students (86% female; 14–20 years, *M* = 16.69, *SD* = 1.04) and *n* = 62 residents (73% female; 67–98 years, *M* = 83.42, *SD* = 7.27). The remaining sample was used as a comparison group without program participation (“control group”): *n* = 345 students (43% female; 14–19 years, *M* = 6.22, *SD* = 0.86) and *n* = 140 residents (70% female; 64–96 years; *M* = 84.79, *SD* = 6.96). There were some demographic imbalances. The proportion of females in the young program group was double the proportion in the young control group, χ^2^(1, *N* = 404) = 37.71, *p* < 0.001, *V* = 0.31. Furthermore, young program group participants were about half a year older than young control group participants, *t*(401) = 3.76, *p* < 0.001, *d* = 0.53. In the older program group, the proportion of lower educated participants was somewhat lower than in the respective control group, χ^2^(2, *N* = 202) = 6.94, *p* = 0.031, *V* = 0.13. However, the program vs. control group difference in cognitive status was only marginally significant, χ^2^(2, *N* = 199) = 5.88, *p* = 0.053, *V* = 0.17; the trend was toward more impairment in the control group.

In the young program group, most participants had zero to three missing sessions (*n*s = 13, 15, 15, and 12, respectively); in the older program group, most participants had zero to two missing sessions (*n*s = 16, 12, and 15, respectively). Only one young participant had more than five missing sessions; in the older program group, this rate was remarkably higher (*n* = 11; these participants did not differ from regular attenders in terms of gender, age, educational level, or cognitive status). In the following, participants who attended less than five (i.e., half of the) sessions were excluded from all analyses, except for the baseline analyses.

### Procedure

#### Baseline Measurement

Participants were first contacted in their classes (students) or at home (residents). They were presented with the LSEP and completed a questionnaire, either in a written form (students) or spoken form (residents), including the question “Are you interested in participating in the LSEP?” (1 = *not at all* to 5 = *very much*) and demographic questions. As part of a multi-measure assessment, this baseline or pre-program (T1) questionnaire also comprised two versions of an age stereotype measure: one version with a focus on one’s own age group (ingroup or self-stereotyping) and another with a focus on the other age group (outgroup or other-stereotyping). In the older participant group, the T1 assessment additionally included a short dementia screening. Informed consent was required from all participants.

#### Program Invitation and Preparation

A couple of weeks later, all students and residents who showed at least some interest in participating in the LSEP (scores ≥ 2; 86% of the students and 80% of the residents) were invited to join an LSEP group. Whether or not they accepted this invitation mainly depended on personal availabilities for the scheduled meeting dates, as respondents’ feedback revealed. Before the LSEP started, the young participants had a preliminary meeting in which they were provided with basic information about human aging, the nursing home as a place to live for older people, and helpful skills for effective intergenerational communication. We thus aimed to prepare the young LSEP participants for their, often first, visit to a nursing home and their encounter with the older participants.

#### Program Participation

Participants of the program group then attended the 10 LSEP sessions on a weekly basis. Due to the older participants’ mobility constraints, the LSEP took place in the nursing homes. If requested by the older participants (e.g., those in a wheelchair or with visual impairments), the adolescent participants provided them with door-to-door transportation service from their apartments to the group meeting place. The group composition of participants and moderators was stable across the 10 sessions. Each session included an evaluation of intergenerational comfort and learning at the session end.

#### Post- and Follow-up Measurements

After finishing the program, participants again completed a questionnaire including an age stereotype measure, this time, however, only with a focus on the other age group (T2; about 3 months after T1). Approximately 3 months after this post-program assessment (and 6 months after T1), participants were asked for a third, follow-up stereotype assessment, corresponding to the T2 procedure (T3). At this measurement point, the questionnaire was also administered to the control group. The lack of a T2 measurement point for the control group was due to practical constraints: the school authority allowed only two measurement points, which each implied a canceled class. Furthermore, a third measurement point for the older participants of the control group would have been difficult to realize, given our and the participants’ limited resources; each participant was individually visited in their apartment and interviewed face-to-face.

**Figure 1:**
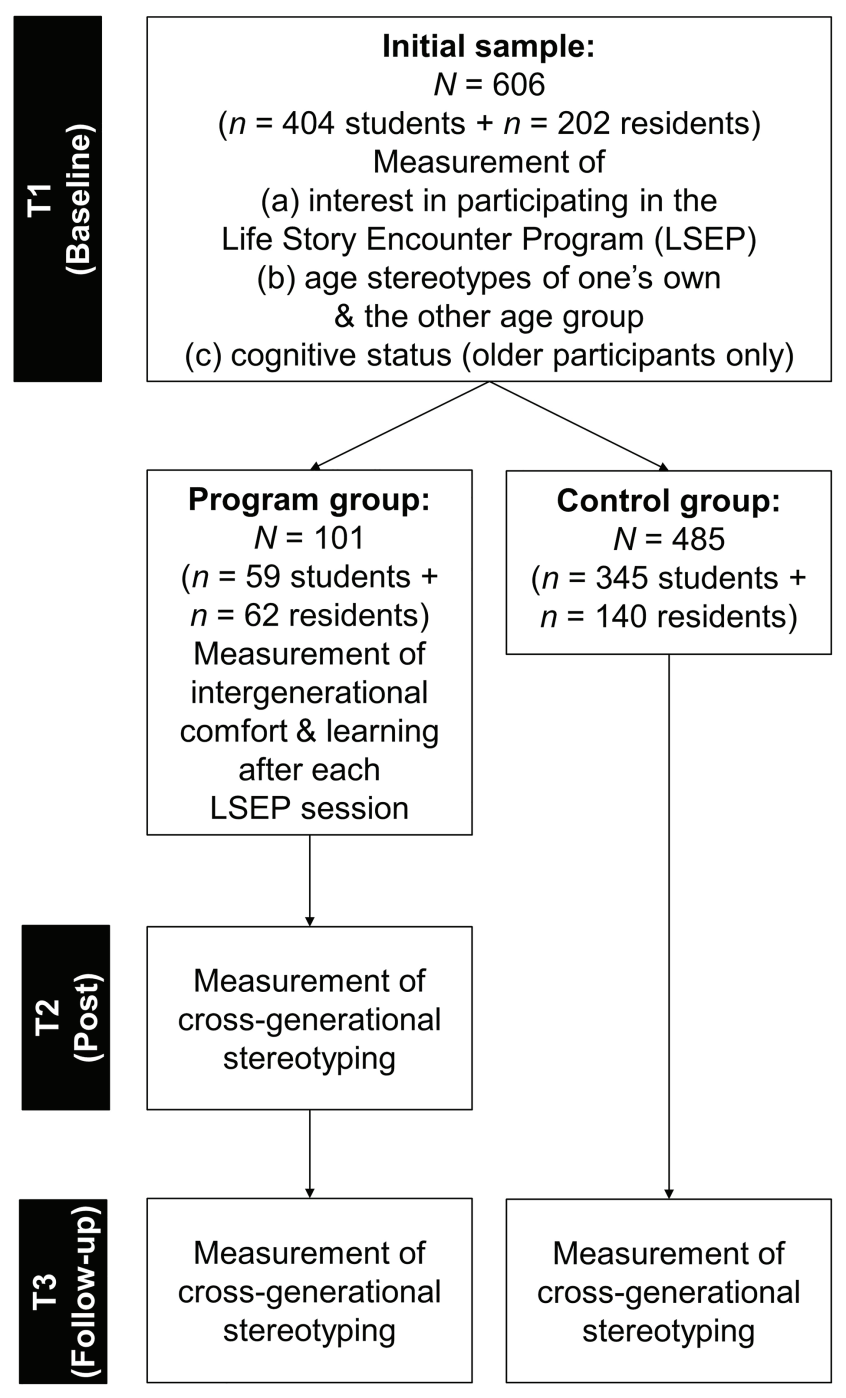
Schematic overview of the study procedure.

Taken together, our study design was quasi-experimental with three measurement points in the program group (T1, T2, and T3) and two measurement points in the control group (T1 and T3). Figure 1 gives a schematic overview of the procedure, including the sample sizes at each measurement point.

### Measures

#### Age Stereotypes

Age stereotypes were measured with the Aging Semantic Differential (ASD; Rosencrantz and McNevin, 1969; German translation by Gluth et al., 2010), one of the most used instruments for the assessment of age stereotypes (for a review, see Ayalon et al., 2019). We chose the ASD for two reasons: first, although developed to measure old age stereotypes, it can be applied to gauge young age stereotypes as well (Gluth et al., 2010). Second, it is an easy-to-understand instrument, even for people who are unfamiliar with completing questionnaires, which applied to most of our older participants.

The original ASD consists of 32 items, covering four age-sensitive trait dimensions, namely, sociability, integrity, instrumentality, and autonomy in the German version. The original 32-item ASD exhausted the older participants at T1; therefore, we had to use a shortened version at T2 and T3. To maintain the broadness of the ASD, we kept those four items of each of the four trait dimensions with the highest loadings. Since, we were not interested in specific age stereotype dimensions but in overall age stereotyping, we combined all items into one scale (for a similar approach, see Couper et al., 1991; Chase, 2011). To allow for meaningful comparisons, the 16-item version was consistently used for all analyses (i.e., across all measurement points, including T1). As the T1 data showed, the shortened ASD correlated very highly with the original, full-length version (*r*s = 0.95 and 0.96 for the young and old age stereotype version, respectively).

As said, at T1, two versions of the ASD were administered to each participant, a young age version and an old age version: “Please indicate how you perceive young people (i.e., adolescents)/older people (i.e., over 70) in general.” Participants responded to each bipolar adjective pair on a seven-point scale (e.g., “Most young/older people are…,” 1 = *unfriendly* to 7 = *friendly*; other sample items were *pessimistic* – *optimistic*, *idle* – *busy*, and *dependent* – *independent*). Higher scores indicate more positive age stereotyping. At T2 and T3, participants were only administered the ASD with the focus on the other generation (i.e., young participants were administered the old age ASD and older participants were administered the young age ASD). Internal consistencies of the ASD measures were 0.83 ≤ αs ≤ 0.89, depending on the target age and measurement point, respectively.

#### Comfort and Learning

After each of the 10 LSEP sessions, participants rated their level of intergenerational comfort (“Did you feel comfortable with each other?”) and learning experience (“Did you learn something important from one another?”) on a five-point scale (1 = *not at all* to 5 = *very much*). According to the group interaction theory by Bales (1950), both processes should be crucial for positive group functioning and, as a consequence and in line with the contact hypothesis by Allport (1954), positive change in CGAS. Although each learning and comfort assessment referred to a specific session and topic, there was a relatively high level of internal consistency across the 10 sessions (αs = 0.74 and 0.87 for comfort and learning, respectively). We thus averaged the comfort and learning measures across the 10 sessions, yielding general measures of intergenerational comfort and learning experience during the LSEP. Both measures were positively interrelated (*r* = 0.46, *p* < 0.001).

### Data Analysis Plan

As a first step, we conducted an analysis of variance (ANOVA) to investigate whether, at T1, the young age stereotype was generally, in the program as well as the control group, more positive than the old age stereotype. Such young age advantage at the baseline measurement point was suggested in Hypothesis 1.

As the second step, we investigated whether participating in the LSEP had a positive impact on participants’ CGAS, as suggested in Hypothesis 2. We conducted two ANOVAs. The first one involved the program group only, namely, changes in CGAS across the three measurements points – before, immediately after, and 3 months after the program (T1, T2, and T3). The second ANOVA included a comparison between the program group and the control group; it was conducted to examine whether changes in CGAS were specific to the program group and absent in the control group. Since we could not assess age stereotypes for the control group at T2, this comparison included only two measurements points (T1 and T3).

As the third step, we conducted a hierarchical regression analysis to investigate whether the hypothesized LSEP-driven change in CGAS resulted from two possible subjective benefits of the encounter program, namely, participants’ social comfort and learning experience during the 10 group sessions. According to Hypothesis 3, both factors should be of predictive value for stereotype change.

## Results

### Baseline Differences in Age Stereotyping

Was the young age stereotype at T1 more positive than the old age stereotype, as suggested in Hypothesis 1? A 2 (stereotype target: young vs. older people) × 2 (age group: young vs. older participants) × 2 (condition: program vs. control group) mixed ANOVA indeed yielded a significant stereotype target main effect, *F*(1, 511) = 65.85, *p* < 0.001, $\eta_{p}^{2}$ = 0.11. As expected, the young age stereotype was more favorable than the old age stereotype (see Table 2 for the descriptives). This medium-sized main effect (Cohen, 1988) was further qualified by a small-sized interaction with age group, *F*(1, 511) = 9.33, *p* = 0.002, $\eta_{p}^{2}$ = 0.02. Bonferroni-corrected pairwise comparisons showed that the advantage of the young age stereotype over the old age stereotype was larger among the older participants than among the young; nevertheless, it was statistically significant in both age groups at *p* < 0.001. Two further, small-sized effects were unrelated to the first hypothesis. A main effect of age group, *F*(1, 511) = 7.70, *p* = 0.006, $\eta_{p}^{2}$ = 0.01, was qualified by an interaction with condition *F*(1, 511) = 8.65, *p* = 0.003, $\eta_{p}^{2}$ = 0.02. The older participants’ more positive age stereotyping was limited to the control condition.

**Table 2:** Descriptives (*M*s and *SE*s) of age stereotyping in the program and control group condition across time (T1, T2, and T3).

Condition	Stereotype target	T1		T2		T3	
Age group		*M*	*SE*	*M*	*SE*	*M*	*SE*
Program group							
Young participants	Young people	4.85	0.07				
	Older people	4.59	0.08	5.04	0.10	4.82	0.11
Older participants	Young people	5.01	0.08	5.37	0.13	5.28	0.10
	Older people	4.42	0.08				
Control group							
Young participants	Young people	4.75	0.04				
	Older people	4.44	0.04			4.34	0.05
Older participants	Young people	5.09	0.07			5.12	0.07
	Older people	4.61	0.12				

Higher scores indicate more positive stereotypes.

Importantly, neither the condition main effect nor its (age-dependent) interaction with stereotype target was statistically significant. That is, at T1, the program and control groups did not differ in their pattern of age stereotyping in either the young or older participants. We additionally performed *t* tests to check more specifically for baseline differences in CGAS, the crucial variable in the following analyses. Confirming the ANOVA results, neither the young program group differed from the young control group in their image of older people, *t*(399) = 1.54, *p* = 0.124, *d* = 0.22, nor did the older program group differ from the older control group in their image of young people, *t*(190) = −0.65, *p* = 0.514, *d* = 0.10.

### Program Effects on Cross-Generational Age Stereotyping

Did the LSEP have a positive impact on participants’ CGAS, as suggested in Hypothesis 2? We conducted two analyses to answer this question, each with a different focus. The first focus was on stereotype change in the program group from T1 to T2 to T3. The second focus was on differences between the program and control group concerning stereotype change from T1 to T3.

The first analysis was a 3 (measurement point: T1 vs. T2 vs. T3) × 2 (age group: young vs. older participants) mixed ANOVA of the CGAS ratings in the program group. Mauchly’s test of sphericity was significant for the stereotype scores, so degrees of freedom were Huynh-Feldt corrected (*ε* = 0.84). As expected, CGAS differed significantly across the three measurement points, *F*(1.69, 155.21) = 13.45, *p* < 0.001, $\eta_{p}^{2}$ = 0.13. All Bonferroni-corrected pairwise comparisons were significant at *p* ≤ 0.03. Irrespective of age group, the post-program stereotypes of the other generation were most positive, followed by the follow-up stereotypes, which were still more positive than the pre-program stereotypes. Reflecting the T1 differences in CGAS, a significant main effect was found for participant age group, *F*(1, 92) = 13.64, *p* = 0.001, $\eta_{p}^{2}$ = 0.13. Across all measurement points, the older participants provided a more positive stereotype of “the young” compared to the adolescents’ stereotype of “the old.”

With regard to the program versus control group comparison, the CGAS ratings were submitted to a 2 (measurement point: T1 vs. T3) × 2 (age group: young vs. older participants) × 2 (condition: program vs. control group) mixed ANOVA. As expected, there was a significant interaction between measurement point and condition, *F*(1, 396) = 15.14, *p* < 0.001, $\eta_{p}^{2}$ = 0.04. Bonferroni-corrected pairwise comparisons showed that only in the program condition, the stereotype of the other generation became more positive from T1 to T3, *p* = 0.001. In the control group, however, CGAS remained stable, *p* = 0.160. Two further effects were significant but unrelated to Hypothesis 2, a main effect of age group, *F*(1, 396) = 60.35, *p* < 0.001, $\eta_{p}^{2}$ = 0.13, and a main effect of condition, *F*(1, 396) = 4.63, *p* = 0.032, $\eta_{p}^{2}$ = 0.01. Again, the young age stereotype was more positive than the old age stereotype; and there were more positive age stereotypes among the program group participants compared to the control group participants.

To compare LSEP effects with other intergenerational programs, we additionally provide effect sizes in terms of standardized mean differences (*d*s). With regard to the T1–T2 and T1–T3 differences in the program group, the effect sizes were *d* = 0.58 (0.73 and 0.49) and 0.38 (0.32 and 0.49), respectively (effect sizes for the young and older program participants in parentheses; computations according to Dunlap et al., 1996). The effect size for the T1–T3 control-group design was *d* = 0.46 (0.54 and 0.40 for the young and older study participants; computations according to Carlson and Schmidt, 1999). According to the classification by Cohen (1988), these effect sizes were small to moderate.

Taken together, the ANOVAs confirmed a generally more negative old age stereotype at T1 compared to the respective young age stereotype. The advantage of the young age stereotype over the old age stereotype was stable across all measurement points. Most importantly, LSEP participation had a substantial impact on CGAS in both age groups. In contrast to the control group, the program group reported more positive CGAS at T2, immediately after LSEP participation, but also at T3, 3 months after LSEP participation. More positive CGAS in the program group applied to both, young participants’ more positive view of older people as well as older participants’ more positive view of young people.

### Pathways Toward Positive Cross-Generational Age Stereotyping

Did the LSEP-driven positive change in CGAS result from two subjective benefits of the encounter program, namely, social comfort and learning experience, as suggested in Hypothesis 3? Before answering this question, it should be noted that participants evaluated the LSEP very positively on both dimensions. The mean ratings of comfort (*M*s = 4.54 and 4.61, *SE*s = 0.04 and 0.06, for the young and older participant group, respectively) and learning (*M*s = 3.71 and 3.99, *SE*s = 0.07 and 0.10) were substantially above the theoretical mean of the one-to-five scale (i.e., the scale point of 3), all *p*s < 0.001.

In an additional 2 (benefit dimension: comfort vs. learning) × 2 (age group: young vs. older participants) mixed ANOVA, both main effects were significant. There were higher comfort than learning scores, *F*(1, 107) = 177.06, *p* < 0.001, $\eta_{p}^{2}$ = 0.62; and older participants reported more benefits than young participants, *F*(1, 107) = 4.36, *p* = 0.04, $\eta_{p}^{2}$ = 0.04.

Our main analysis then consisted of two hierarchical regression analyses. We regressed participants’ CGAS at T2 (post-program measurement; first analysis) and at T3 (follow-up measurement; second analysis) on the respective measure at T1 (the autoregressor), comfort and learning during the LSEP plus their interaction (Block 1), and age group effects plus age moderated effects of comfort, learning, and their interaction (Block 2). The variables entered in Block 1 thus differentiate between stability of CGAS (from T1 to T2 and to T3, respectively) and changes thereof due to perceived program benefits (comfort and learning). The variables entered in Block 2 indicate, besides age group differences, possible age-dependent program benefits. The variance inflation indicators (VIFs ≤ 2.04) showed that multicollinearity was no problem in the regression analyses. Results are presented in Table 3.

**Table 3:** Hierarchical regressions (*B*s and *SE*s; total and incremental *R*s) of cross-generational age stereotypes at T2 and T3 on cross-generational age stereotypes at T1, program benefits (Block 1), age group, and age moderated program benefits (Block 2).

	Cross-generational age stereotype T2				Cross-generational age stereotype T3			
	Block 1		Block 2		Block 1		Block 2	
	*B*	*SE*	*B*	*SE*	*B*	*SE*	*B*	*SE*
Cross-generational age stereotype T1	0.10	0.12	0.01	0.13	0.33^**^	0.12	0.26^*^	0.12
Comfort	0.34	0.23	0.46	0.24	0.44	0.23	0.57^*^	0.22
Learning	0.36^**^	0.13	0.28^*^	0.14	0.06	0.14	0.01	0.13
Comfort × Learning	−0.80^**^	0.31	−0.53	0.39	0.59	0.39	0.73	0.39
Age group			0.20^*^	0.09			0.12	0.08
Comfort × Age group			−0.25	0.23			−0.57^**^	0.21
Learning × Age group			0.06	0.14			−0.20	0.13
Comfort × Learning × Age group			−0.64	0.39			0.08	0.40
*R*^2^_Incremental_			0.04				0.16^***^	
*R*^2^_Total_	0.24^*^		0.28^***^		0.16^**^		0.32^***^	

* *p* < 0.05;

** *p* < 0.01;

*** *p* < 0.001.

**Figure 2:**
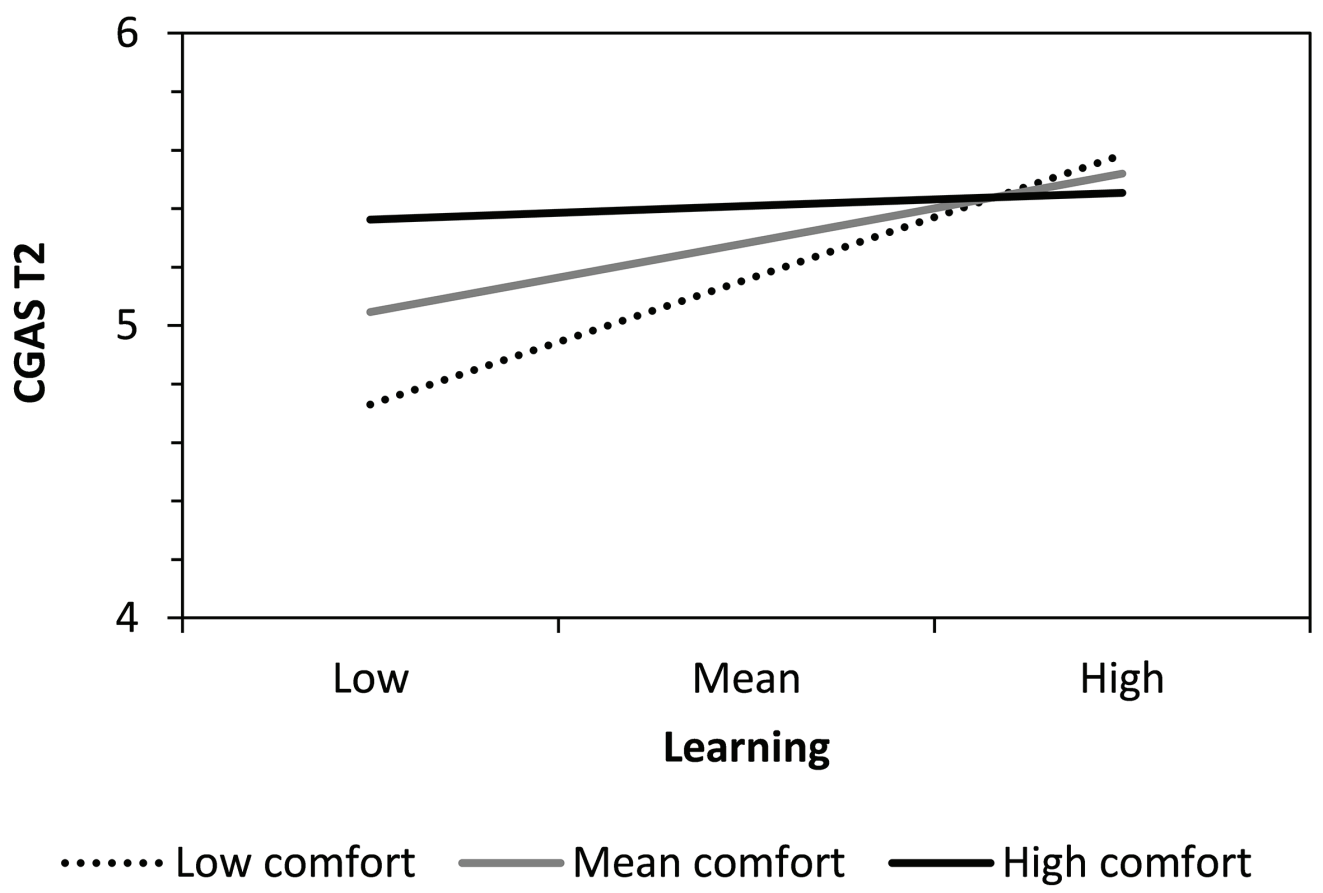
Comfort-moderated effects of learning experience during the LSEP on T2 cross-generational age stereotyping (CGAS; after controlling for autoregressive T1–T2 effects).

The first regression examined changes in CGAS from T1 to T2 (pre/post comparison). Block 1 showed a significant effect of learning experience. Participants who benefited from intergenerational learning during the program displayed a more positive stereotype of the other generation at the end of the program. This effect was further moderated by comfort. To understand this interaction, we performed a simple slopes analysis and plotted the regression of T2 stereotyping (when controlling for autoregressive T1–T2 effects) on learning experience at low, mean, and high levels of social comfort (see Figure 2). Learning only had a significant positive effect on T1– T2 stereotype change when comfort was relatively low (1 *SD* below mean), *B* = 0.66, *SE* = 0.16, *p* < 0.001, or mean, *B* = 0.34, *SE* = 0.13, *p* = 0.012, but not when comfort was high (1 *SD* above mean), *B* = 0.02, *SE* = 0.20, *p* = 0.927. The predictors entered in Block 2 did not significantly explain additional variance in T1–T2 stereotype change.

**Figure 3:**
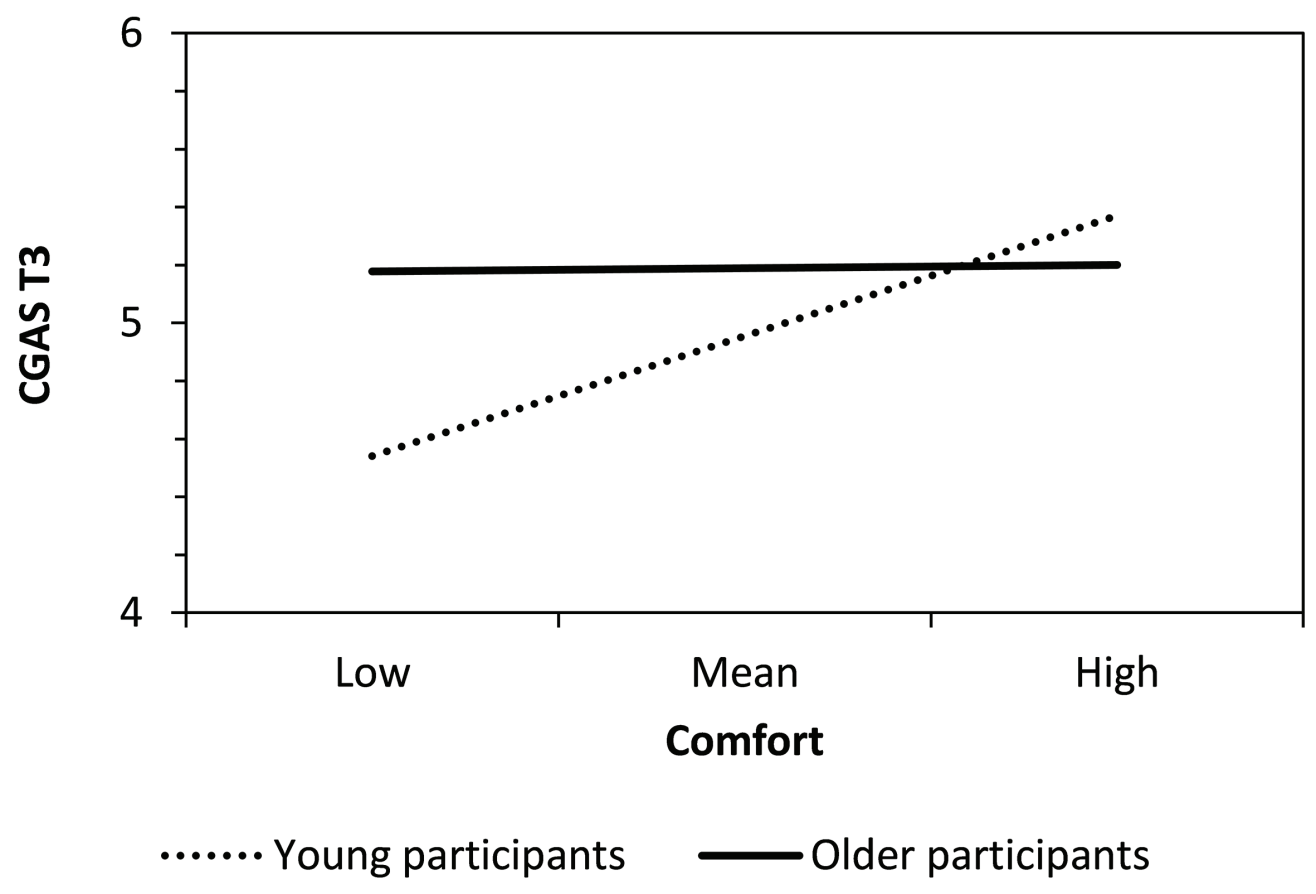
Age-moderated effects of social comfort during the LSEP on T3 CGAS (after controlling for autoregressive T1–T3 effects).

The second regression examined changes in CGAS from T1 to T3 (pre/follow-up comparison). In contrast to the first regression, there was substantial stability between T1 and T3 stereotyping (the autoregressor turned out to be statistically significant). No other Block 1 predictor was significant. This time, the variables entered in Block 2 contributed to explaining additional variance in T1–T3 stereotype change. There was a significant effect of comfort (it was only marginally significant in Block 1). Participants who reported a higher level of comfort during the program showed a more positive stereotype of the other generation at T3. This effect was moderated by age group. A simple slopes analysis (see Figure 3) showed that, only for adolescents, comfort had a significant positive effect on T1–T3 stereotype change, *B* = 1.13, *SE* = 0.33, *p* < 0.001, but not for the older participants, *B* < 0.01, *SE* = 0.28, *p* = 0.996.

Taken together, the regression analyses confirmed Hypothesis 3 by showing that perceived social comfort and learning during the program contributed to predicting positive change in CGAS from T1 to T2 and to T3, respectively. Specifically, subjective comfort compensated for the negative impact of little learning experience on T1–T2 stereotype change, and comfort predicted T1–T3 stereotype change in the younger participant sample.

### Additional Control Analyses

Participants could not be randomly assigned to the program vs. control group conditions. Apart from personal availability for the scheduled meeting dates, participation in the LSEP mainly depended on personal interest in the program. In other words, our findings might reflect a self-selection bias. Therefore, we repeated all analyses and included LSEP interest as a control variable. The pattern of results remained unchanged. We also conducted an intention-to-participate analysis, analogous to an intention-to-treat analysis. That is, we analyzed whether the positive change in CGAS persisted, if participants with less than five sessions attended were kept in the analyses (in the previous analyses, they were excluded). Indeed, this was the case, speaking against a positivity bias due to non-random attrition effects. Consistent with this, frequency of participation was unrelated to any of the demographics or older participants’ cognitive status.

Furthermore, gender, age, and, in the older participant group, educational level and cognitive status might have had an impact on findings. Demographics were unbalanced in some subgroups and the older participant group included, according to our dementia screening, a considerable number of participants with mild cognitive impairment or early dementia. Therefore, we also tested, in the young and older groups separately, whether these variables were associated with age stereotyping at any of the measurement points or with program evaluation (intergenerational comfort and learning). This, however, was not the case, in either the program or, where applicable, in the control group. Results of the additional analyses can be obtained from the first author upon request.

## Discussion

Intergenerational solidarity is a core element of social functioning and community building (Bengtson and Oyama, 2010). Bringing young and older people together to strengthen intergenerational relationships beyond the familial context thus makes intuitive sense. That said, existing intergenerational programs have often been criticized for two reasons: lacking theoretical foundation and limited evaluation research (Abrams and Giles, 1999; Jarrott, 2011; Kuehne and Melville, 2014). The present study addressed these critical issues and aimed to investigate the impact of a newly developed intergenerational program on CGAS.

Based on a biographical-narrative approach, the LSEP provides adolescent and older participants the opportunity to share existential questions of life – issues that should be of general human concern, regardless of age or any other demographic. Specifically, we translated the LSI (McAdams, 2007) into a group format. Referring to the intergroup contact hypothesis (Allport, 1954), we suggested that participating in the LSEP should have a sustainable positive impact on CGAS. The LSEP was piloted in a nursing home setting using secondary school students and residents as participants. Our intervention study design was longitudinal and included a control group without LSEP participation.

### Positive LSEP Effects on Cross-Generational Age Stereotyping

At the first, baseline measurement point (T1), we corroborated, in line with Hypothesis 1, an asymmetry that has often been found in previous age stereotype research (Kite et al., 1991; Hummert et al., 1995; Grühn et al., 2011; Ng et al., 2015): a more positive young age stereotype compared to the old age stereotype. This asymmetry emerged in the program as well as the control group. Interestingly, older participants reported an even greater advantage of the young age stereotype over the old age stereotype than young participants did. Whether this reflects older people’s “sentimental journey” to their youth (Wong and Watt, 1991; Coleman, 2005; Hepper et al., 2020) or, more detrimentally, the incorporation of a negative old age stereotype into their self-concept (*internalized ageism*; Levy, 2009; Bennett and Gaines, 2010; Kornadt et al., 2017) remains an open question.

When comparing pre/post CGAS in the program group, we found, in line with Hypothesis 2, that both adolescent and older participants reported a more positive stereotype of the other generation after the program end. Importantly, this effect faded somewhat, but was still detectable, at the follow-up measurement point, 3 months after the program end. The comparison between the program group and the control group, for which we only had the baseline and follow-up stereotype measures, also confirmed Hypothesis 2. The positive change in CGAS was restricted to the program group; in the control group, however, CGAS remained stable over the 6-month period.

According to the classification by Cohen (1988), the LSEP effects on CGAS were small- to medium-sized, depending on the time interval and age group. Unfortunately, we could not compare our effect sizes with those reported in the four reference studies (Pinquart et al., 2000; Meshel and McGlynn, 2004; Belgrave, 2011; Gamliel and Gabay, 2014), since these did not provide the necessary statistics. The mean effect sizes of anti-ageism programs, as reported in meta-analyses by Beelmann and Heinemann (2014; *d* = 0.38) and Burnes et al. (2019; *d* = 0.33), were somewhat smaller than the corresponding LSEP effect size (*d* = 0.56). The LSEP advantage might be even larger when considering that almost all of the studies reported in the two meta-analyses used a pre/post control-group design, whereas our effect size refers to a control-group design that compared a pre measurement point with a more distant follow-up measurement point. Nevertheless, we agree with Burnes et al. (2019) that anti-prejudice programs are relatively low-cost, feasible strategies to reduce ageism, and we share the position of Beelmann and Heinemann (2014) that even small effects of anti-prejudice programs can have important practical significance with regard to discrimination reduction.

Our results confirmed the effectiveness of the LSEP in promoting positive age stereotypes through intergenerational contact and substantiated the contact hypothesis by Allport (1954) in the context of a newly developed intergenerational program. Importantly, stereotype change could be found for the adolescents’ old age stereotype as well as the older people’s young age stereotype. Compared to extensive evidence about young people’s mostly negative views of “the old” (e.g., Williams et al., 1997; Cuddy et al., 2005; Kite et al., 2005; Nelson, 2005), there is little research on the attitudes older people have toward young people (for exceptions, see Matheson et al., 2000; Chasteen, 2005) – and particularly little research on the effects of intergenerational programming on older people’s young age stereotypes. The present study contributes to filling this gap.

### Intergenerational Comfort and Learning as Crucial Processes

Although there is plenty of evidence for the contact hypothesis by Allport (1954), processes often remain unclear (Pettigrew and Tropp, 2008; McKeown and Dixon, 2017). Referring to the group interaction theory by Bales (1950), we hypothesized two processes that should account for the impact of LSEP participation on CGAS: a social-emotional one (feeling comfortable with each other) and a task-related one (learning from one another). Indeed, our analyses showed that both pathways contributed to predicting positive stereotype change in the pre/post as well as the pre/follow-up interval (i.e., from T1 to T2 and to T3, respectively). Learning was more important to T1–T2 stereotype change, while comfort was more important to T1–T3 stereotype change. The advantage of intergenerational comfort over learning effects with regard to the long-term amelioration of CGAS might reflect the primacy of the relationship vs. content aspect in communication theory (Watzlawick et al., 1967) or the primacy of the communion vs. agency dimension in impression formation research (Abele and Wojciszke, 2007). Furthermore, there was a complex interplay of both program benefits. Subjective comfort compensated for the negative impact of little subjective learning experience on T1–T2 stereotype change. This interaction effect substantiates the idea of Bales (1950) that social-emotional and task-related processes mutually reinforce each other and thus are indispensable to positive group functioning.

Our results are fully compatible with the recently published Positive Education about Aging and Contact Experiences (PEACE) model by Levy (2018). Referring to correlational and experimental research on (age) stereotyping and relevant theoretical work, the author proposes two key factors that can reduce ageism in everyday life: (a) education about aging, including facts on aging along with positive older role models; (b) positive contact experiences with older people, involving the sharing of personal information (e.g., significant life events and life lessons). Levy (2018) called for putting the model into practice by creating specific intervention programs and conducting respective intervention research. We think the present study has unintentionally (we developed the LSEP before Levy’s publication has been released) answered this call, and provides solid evidence for the PEACE model.

Two further results, though not directly related to our hypotheses, deserve some attention. Firstly, the LSEP was evaluated very positively in terms of both learning experience and, especially, social comfort. Thus, the program is not only effective in promoting more favorable CGAS; but also it is esteemed by participants as personally beneficial. That the older participants reported high social comfort after the sessions is in line with research on the positive impact of reminiscence interventions on subjective well-being (Pinquart and Forstmeier, 2012; Gaggioli et al., 2014), especially when such interventions are conducted in group settings (Haslam et al., 2010, 2014). Interestingly, older participants reported more learning progress than young participants did. This result contradicts a prominent age stereotype, according to which “the young” are open and “the old” resistant to change, the latter due to lowered or even lost learning capacity and willingness (Wrenn and Maurer, 2004; Brooke and Taylor, 2005).

Second, referring to correlations between measurement points, we found no differential stability in age stereotyping from T1 to T2, but substantial differential stability from T1 to T3. We interpret this finding in the sense of a fading but not disappearing impact of LSEP participation on age stereotyping (see Bailey et al., 2017, for fadeout effects in intervention research). In other words, the more time passes by after LSEP termination, the less intergenerational contact participants might have, the more negative aspects of their original image of the other generation might return. Admittedly, this assumption needs future investigation.

### Theoretical and Practical Implications

The present study has important theoretical and practical implications. Our focus was on cross-generational young and old age stereotyping. This is consistent with a greater understanding of age stereotypes as overgeneralized beliefs about people belonging to a particular age group, but deviates from developmental research that equates age stereotypes with old age stereotypes (e.g., Hummert, 1990; Kessler and Staudinger, 2007). We think that a greater understanding of age stereotypes broadens the perspective to any prejudice and discrimination based on age, including ageism directed to younger people (*adultism*; Flasher, 1978; Bell, 2010).

Our study directs the attention toward the stability vs. changeability of age stereotypes. Since age is a primary dimension of interpersonal categorization (North and Fiske, 2012) and ageism is prevalent in Western societies (Hummert, 2011), it is not surprising that negative old age stereotypes already exist in preschool children and then stabilize across middle childhood and adolescence (Davidson et al., 2008; Flamion et al., 2020). Nevertheless, age stereotypes are changeable, as the present study underlines (see also Meshel and McGlynn, 2004; Gamliel and Gabay, 2014). Changing negative age stereotypes promotes intergenerational relationships (Hummert et al., 2004), but is also important from an individual developmental perspective. Older people form the only outgroup that, inevitably, becomes one’s ingroup, as one ages. Thus, negative old age stereotypes are likely to obstruct, in the sense of self-fulfilling prophecies, positive development in older age (Lamont et al., 2015).

The LSEP content itself is likely to contribute to participants’ positive development. Referring to the stages of psychosocial development by Erikson (1959), sharing one’s thoughts about existential questions on the basis of individual life stories should support the young participants in developing their identity, a primary developmental task in adolescence. With regard to the older participants, the LSEP should provide the opportunity to satisfy generative needs, a primary developmental task in late adulthood. Nevertheless, it would be wrong to describe the LSEP as a one-way program of advice giving and receiving. As our results show, both generations reported high levels of mutual learning (and social comfort) during the 10 LSEP sessions.

This leads to our last point. In their review of the role that reciprocity plays in intergenerational programs for adolescents and older adults, Knight et al. (2014, p. 275) conclude “future research needs to ensure intergenerational interventions explicitly involve reciprocity of giving, and directly measure the psychosocial benefits for both generations.” The present study verifies reciprocity is of core importance in intergenerational programs involving adolescents. As our results show, older LSEP participants feel comfortable with young participants and vice versa, and young participants learn from the older ones and vice versa. Mutual giving and receiving, or, put differently, the interplay between generosity and gratitude, allows interaction on an equal level. And such interaction has the power to reduce mutual stereotyping.

## Limitations and Outlook

Several limitations of the present study should be considered. The sample size was limited for practical reasons: organizing a 10-session encounter program for 12 intergenerational groups who meet in different nursing homes is a major logistical challenge. Nevertheless, a larger sample size would have allowed for more complex analyses (e.g., analyses that take the multilevel structure of the data into account). The sample we used was not representative of the German young and older population, respectively. The adolescents were better educated than average, and the nursing home residents probably had a lower health status than most people their age. It is remarkable that the present study confirmed LSEP effects on age stereotyping in both age groups, though the older participants’ cognitive status was lower than expected. Consistently, older participants’ stereotyping was unrelated to their cognitive status. This pattern is in line with prior findings on the effectiveness of biographical-narrative programs for older people with early dementia (Giraudeau and Bailly, 2019). At what stage dementia becomes an exclusion criterion for LSEP participation due to little or no benefits, or even excessive demands, deserves further research.

We do not disregard the main methodological limitation of our study, namely non-randomization. Therefore, a future study might apply a waiting list design, using two randomly assigned groups with equal levels of interest in LSEP participation. One group, the waiting list control group, serves as an “untreated” comparison group during a first study phase, but then continues with LSEP participation in the second study phase. Complete denial of LSEP participation would be ethically questionable. Realizing a waiting list design with older participants, however, can also be ethically problematic, as waiting might be especially unpleasant when individuals’ future perspective is uncertain (Pierce et al., 2019). Given the limitation of non-randomization, we made some effort to control for both non-random assignment and attrition effects. We showed that (a) stereotype change effects were stable when controlling for interest in LSEP participation; (b) participants excluded from the main analysis due to low program attendance did not differ in demographics or cognitive status; (c) stereotype change effects were robust when (re-) including participants with low program attendance (intention-to-participate analysis); and (d) stereotyping was unrelated to program vs. control group membership, demographics, and cognitive status.

Although the shortened stereotype scale we used showed acceptable reliability as a measure of general age stereotyping, it did not allow for the detailed, reliable measurement of different stereotype domains. Program benefits were measured after each session with single items, of which the reliability is always questionable. Nevertheless, there was remarkable consistency in social comfort and learning experience across the 10 group sessions. Furthermore, we cannot rule out that the older participants reported more learning experience than the young because the former have a broader, holistic understanding of learning; adolescents might cut down learning to acquiring formalized (vs. experiential) knowledge, which is (all too) typical to the school context (Holzkamp, 1993). It would be of particular interest to assess in future studies what exactly participants learned from each other during the program (e.g., insight in life course dynamics and different living contexts, ways of coping with life adversities; oral history, value orientations, and communication skills) or what exactly contributed to intergenerational comfort. To this end, it might be advantageous to combine quantitative and qualitative evaluation methods. For example, open-ended interviews or group discussions could be adequate formats to evaluate participants’ LSEP-related feelings, reflections, and insights.

Before the LSEP started, only the young participants had a preparatory meeting in which they were provided with basic information about aging. We cannot rule out that this meeting had a specific effect on young LSEP participants’ old age stereotyping. Two reasons speak against this, however: first, the preparatory meeting lasted only 90 min, whereas the LSEP consisted of 10 90-min sessions on a weekly basis. Second, the information provided to young LSEP participants focused, due to practical reasons, on older people’s mobility, auditory, and speech constraints – and how to deal with these. If the preparatory meeting had an impact on young LSEP participants’ old age stereotype, this should have been rather negative than positive. The contrary, however, was the case.

Finally, with regard to the general applicability and effectiveness of the LSEP, it would be of great interest to evaluate the program in other settings than nursing homes, such as school contexts, community centers, or gerontological education programs. It would be of further interest to see whether the LSEP works with people in middle adulthood as well as with adolescents. The “sandwich generation” is mostly forgotten in intergenerational programming (MacCallum et al., 2006). It might also be worth thinking about combining face-to-face and virtual elements within the LSEP; a recent review showed that such combined intergenerational programs are as effective as conventional face-to-face programs (Canedo-García et al., 2017). A virtual mode of the LSEP might be of special value if participants cannot physically meet, as is the case for the current COVID-19 pandemic with its heightened risk of ageism (Ayalon et al., 2020). We are optimistic that the program effects we found with regard to CGAS generalize across different settings, ages, and modalities. Whether they generalize across diverse cultural contexts, with their specific age norms, is an open and intriguing question (Lou and Dai, 2017).

## Conclusion

The present study showed that bringing together secondary school students and nursing home residents to commonly reflect upon and talk about existential questions of life, based on their personal life stories, was to the benefit of both generations. Young as well as older LSEP participants felt comfortable with and learned from each other, which predicted positive CGAS. Such change could not be observed in the control condition.

At first sight, these results might be considered as not surprising, since the LSEP is a theoretically well founded intergenerational program, based on the intergroup contact hypothesis by Allport (1954), the small group research by Bales (1950), and the developmental concepts of identity formation and generativity by Erikson (1959). Indeed, “there is nothing so practical as a good theory,” to quote a famous dictum by Lewin (1951, p. 169). In other words, a good theory is necessary but not sufficient for good practice; what further counts is its appropriate application and implementation. This, however, is far from trivial and requires sound evaluation research, which we tried to conduct in the present study. We think the results are convincing and promising.

In our view, intergenerational programs, such as the LSEP, are of particular relevance in aging, but increasingly age-segregated societies. Virtually every country in the world is experiencing growth in the number and proportion of older persons in their population (United Nations, 2019). At the same time, however, the socio-spatial separation of the generations is deepening, especially in the Western world (Hagestad and Uhlenberg, 2006). Against this backdrop, the LSEP is timely and important; it has the potential to foster ties between the young and older generation beyond the familial context.

## Data Availability Statement

The raw data supporting the conclusions of this article will be made available by the authors, without undue reservation.

## Ethics Statement

The studies involving human participants were reviewed and approved by Aufsichts- und Dienstleistungsbehörde Rheinland-Pfalz (the administrative authority of the federal state of Rhineland-Palatinate responsible for social affairs and education). Written informed consent from the participants’ legal guardian/next of kin was not required to participate in this study in accordance with the national legislation and the institutional requirements.

## Author Contributions

All authors listed have made a substantial, direct and intellectual contribution to the work, and approved it for publication.

### Conflict of Interest

The authors declare that the research was conducted in the absence of any commercial or financial relationships that could be construed as a potential conflict of interest.
